# Bibliography

**Published:** 1905-01-15

**Authors:** 


### BIBLIOGRAPHY.

ORTHODONTIA AND ORTHOPEDIA OF THE FACE. This is a new work by Victor H. Jackson, of New York, which is just published by the J. B. Lippincott Co., of Philadelphia. The work contains over five hundred pages and is fully illustrated with over seven hundred original drawings. The principal interest in this work centers about the fact that it sets forth in a most comprehensive manner a unique system of correcting mal-occlusion of the teeth. The basis of the system is the method of attaching the appliances used for applying the force. This is by means of a wire "crib" usually attached to the molar or bicuspid teeth, and made of springy wire. To this crib are attached wire levers by means of soft solder which are used to apply a constant pressure on the teeth to be moved. The appliances are removable and adjustable. The claim of the author is that the so-called "Labor and Rest" appliances, such as the Angle system, because they operate by producing periods of irritation and rest alternately are likely to produce increased growth or hypertrophy of the tissues, while constant pressure, will produce absorption, which is desirable in order that the teeth may be changed in position, particularly if the anchorage be so secure that the force or pressure is constant and not vacillating, as is the case with many methods used in correcting irregularities of the teeth. The great variety of cases reported in the book and the successful issues certainly indicate that the author has good reason for the confidences exhibited in this book which has cost him years of time and large expense.

There are many statements in the book that one feels he would like to have verified, but which, unless he has used the system, he does not feel qualified to contradict. The illustrations are there to prove the statements and they cannot therefore be questioned. There is one criticism we would make of the illustrations for future additions. There are too many of them, and the point always made is to show the appliance rather than the case. The drawing of the appliances are perfectly intelligible in almost every case, but the teeth in the majority of the illustrations are so poorly drawn that in some cases it is difficult to make a correct diagnosis; this is especially true where but one jaw is shown in the illustration. We do not see how one can determine the treatment of a case of mal-occlusion of the teeth without good plaster models for study and reference, and by good models we mean as nearly perfection as is practicable to obtain with the most accurate impression material. Perhaps this criticism is not pertinent, because this book is intended to set forth a system of appliances and methods; but in the preface the author indicates that he anticipates that the work may be used as a text book in our colleges. To be successfully used in the class room it must embrace all the fundamental principles and show its adaptation in a clear and comprehensive manner. The book is full of suggestions for treating cases as they have occurred in the author's practice and as they are liable to occur in any general practice, and we predict that the practitioners will appreciate the work Dr. Jackson has so conscientiously done more than students or teachers. The work is timely and well done, and the book ought to be in the library of every dentist who attempts to do this kind of work. The subject of Orthodontia is more prominently before the profession at this time than any other, and it will increase any practitioner's ability to master this subject if he shall read this book. It is very entertainingly written.

